# A Tug of War: DNA-Sensing Antiviral Innate Immunity and Herpes Simplex Virus Type I Infection

**DOI:** 10.3389/fmicb.2019.02627

**Published:** 2019-11-26

**Authors:** Yingying Lin, Chunfu Zheng

**Affiliations:** Department of Microbiology, Immunology and Infectious Diseases, University of Calgary, Calgary, AB, Canada

**Keywords:** herpes simplex virus type I, DNA sensor, immune evasion, interferon, antiviral immunity

## Abstract

Cytosolic DNA sensors are the most recently described class of pattern recognition receptors (PRRs), which induce the production of type I interferons (IFN-I) and trigger the induction of a rapid and efficient innate immune response. Herpes simplex virus type I (HSV-1), a typical DNA virus, has displayed the ability to manipulate and evade host antiviral innate immune responses. Therefore, with an aim to highlight IFN-I-mediated innate immune response in a battle against viral infection, we have summarized the current understandings of DNA-sensing signal pathways and the most recent findings on the molecular mechanisms utilized by HSV-1 to counteract antiviral immune responses. A comprehensive understanding of the interplay between HSV-1 and host early antiviral immune responses will contribute to the development of novel therapies and vaccines in the future.

## Introduction

Herpes simplex virus type I (HSV-1), a member of the alphaherpesvirus subfamily, has already co-evolved with human beings for thousands of years and is well known for its high prevalence in the population worldwide (Davison, 2010; Sharma et al., 2016; Ibanez et al., 2018). With a large linear double-stranded DNA that encodes over 80 proteins, HSV-1 can produce lifelong infections in the host, and this is achieved thanks to its capacity to infect epithelial cells, neurons, and other cell types, including immune cells *in vivo* and *in vitro* (Wu et al., 2016; Koelle et al., 2017; Tognarelli et al., 2019).

Clinically, HSV-1 is mainly associated with orofacial lesions, yet it is also the leading cause of infectious blindness in developed countries and viral encephalitis in adults (Horowitz et al., 2010; Farooq and Shukla, 2012; Bernstein et al., 2013). After the initial infection, HSV-1 becomes latent in the trigeminal ganglion, and recurrent reactivation leads to different immunopathology, which may cause neuronal damage and Alzheimer’s disease (AD) (Devanand, 2018).

Early detection of viral invasion by pattern recognition receptors (PRRs) is crucial for the induction of a rapid and efficient innate immune response. Cytosolic DNA sensors are the most recently described class of PRRs, which also include the Toll-like receptors (TLRs), certain RNA sensors, such as RIG-I-like receptors and melanoma differentiation-associated gene 5 (Wu and Chen, 2014; Su et al., 2016). Viral nucleic acids of HSV-1, recognized by various PRRs, can act as strong activators of various signaling pathways that finally promote antiviral immune responses through the secretion of pro-inflammatory cytokines, as well as the production of type-I interferons (IFN-I) in infected cells (Iwasaki, 2012). The activation of the IFN-I pathway ultimately induces the expression of multiple IFN-stimulated genes (ISGs) and boosts the innate immune responses (Schoggins, 2019). HSV-1 has been reported to evade host immunity and facilitate its infection and replication through multiple strategies (Schulz and Mossman, 2016; Christensen and Paludan, 2017).

Although different cytosolic DNA-sensing pathways can be activated, HSV-1 has developed multiple mechanisms to attenuate this host antiviral machinery (Zheng, 2018). In this review, we outline the recent findings with the aim of highlighting antiviral innate immune responses in the battle against the HSV infection. A comprehensive understanding of the interplay between HSV-1 and host antiviral innate immunity could contribute to the development of novel immunotherapies and effective vaccines to counteract this virus over the next few decades.

## Interplay Between the Host Antiviral DNA-Sensing Pathways and Herpes Simplex Virus Type I

The newly emerging DNA in the cytoplasm induces robust and rapid innate immune responses through its binding to various DNA sensors, including TLR9, absent in melanoma 2 (AIM2), RNA polymerase III, Interferon-γ inducible protein 16 (IFI16), DEAD-box helicase 41 (DDX41), and some proteins involved in the DNA damage responses, among which the cyclic guanosine monophosphate-adenosine monophosphate synthase (cGAS) is the only one that has been identified as a universal cytoplasmic DNA sensor in various cell types (Lund et al., 2003; Chiu et al., 2009a; Zhang et al., 2011; Sun et al., 2013; Zheng, 2018; Stempel et al., 2019). TLRs have been described to mediate antiviral activities against HSV during infection. If the animals lacked both TLR2 and TLR9, all animals were more susceptible to infection than single knockout animals pointing out the relevance of these receptors during HSV infection (Lima et al., 2010; Uyangaa et al., 2018). Furthermore, HSV-1 infection in human neurons was shown to be suppressed by type-III IFN (IFN-λ) through the upregulation of TLR9 expression and subsequent TLR9-mediated antiviral responses involving the transcription factor interferon regulatory factor 7 (IRF7) (Zhou et al., 2011). But this result remains to be determined because IFN-λ has been reported to be secreted during HSV infection in the vaginal mucosa, mainly by dendritic cells (Iversen et al., 2010). Although AIM2 also detects aberrantly localized DNA, it is currently proposed that it cooperates with IFI16 and activates the inflammasome (Lugrin and Martinon, 2018). Other proposed DNA sensors, such as DDX41, also require further investigation to clarify their role during HSV infection and if they act redundantly in a cell-type-dependent manner (Zhang et al., 2011). Furthermore, unlike cGAS and IFI16, these sensors have, thus far, not been shown to restrict the replication of HSV-1 and have been evaded by HSV-1.

**Figure 1:**
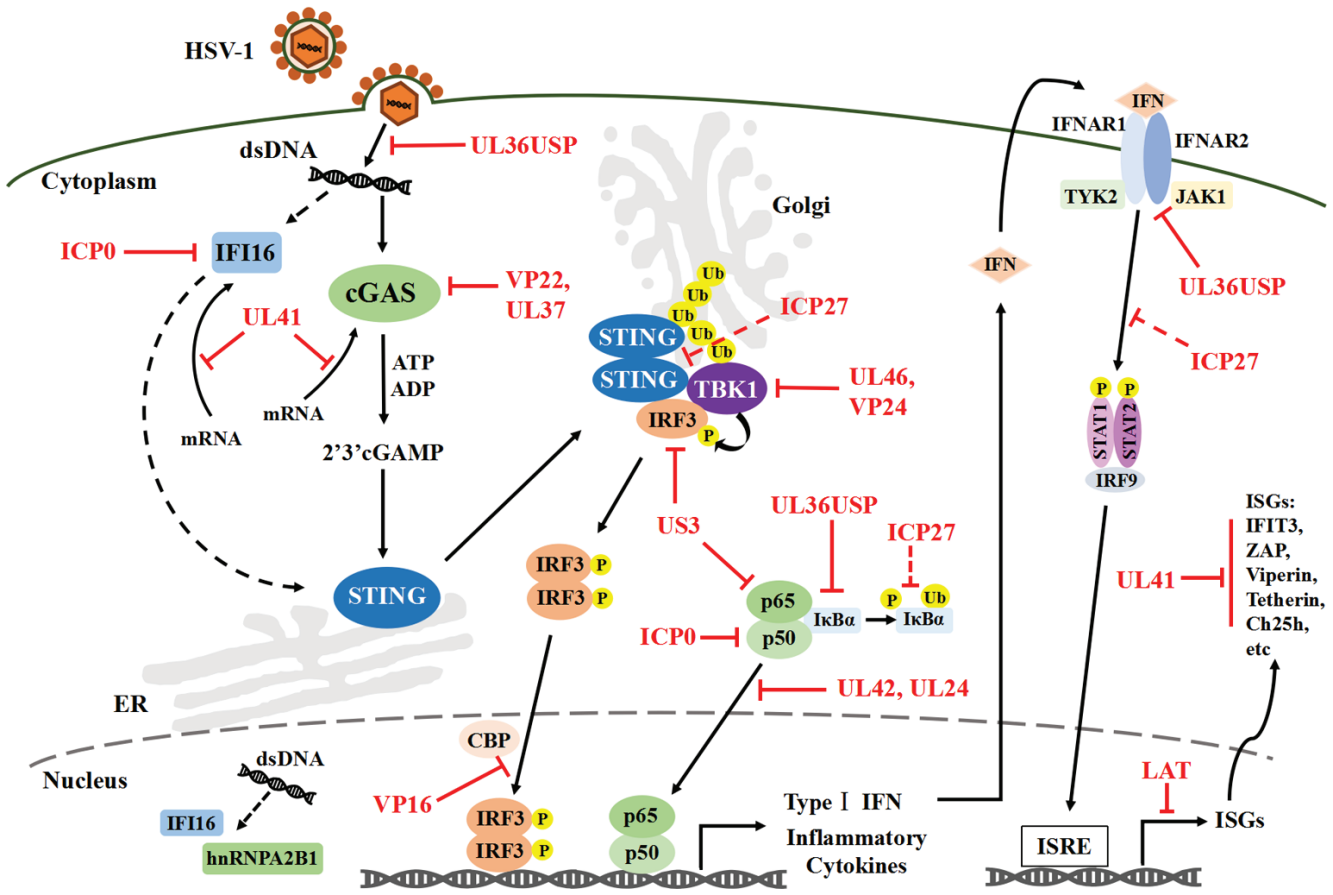
HSV-1 mediated evasion of the DNA-sensing pathway in innate immunity. Cytosolic DNA sensors, such as IFI16 and cGAS, can recognize HSV-1 dsDNA and trigger IRF3 and NF-κB activation, which results in the production of IFN-I and antiviral immune responses. Multiple steps in the DNA-sensor-mediated IFN-I signaling pathway can be targeted by HSV-1 proteins, including cGAS-mediated viral recognition and subsequent signaling pathways. Solid lines indicate confirmed interactions between the host signaling proteins and HSV-1 proteins. Dashed lines indicate uncertain interactions. HSV-1, Herpes simplex virus type I; IFI16, Interferon-γ inducible protein 16; cGAS, Cyclic guanosine monophosphate-adenosine monophosphate synthase; dsDNA, Double-stranded DNA; IRF3, Interferon regulatory factor 3; NF-κB, Nuclear factor κB; IFN-I, Type I interferons; P, Phosphate; Ub, Ubiquitin.

In this review, specific attention is given to the cGAS-STING DNA-sensing signal pathways and its downstream IFN-I signal pathway, which plays a central role in innate antiviral immunity (Figure 1).

### Interferon-γ Inducible Protein 16

IFI16 is a host sensor of nucleic acids that has been reported to recognize cytosolic double-stranded DNA (dsDNA) as well as HSV-1-derived DNA in the nucleus (Unterholzner et al., 2010). HSV-1 recognition by IFI16, which itself results in acetylation and redistribution to the cytoplasm, then induces the activation of the transcription factor interferon regulatory factor 3 (IRF3) and transcription factor’s nuclear factor κB (NF-κB) into the nucleus (Pilli et al., 2012; Ansari et al., 2015). These processes are followed by the production of IFN-I and IL-6, which are able to restrict viral replication and initiate an inflammatory response (Conrady et al., 2012; Ansari et al., 2015; Zheng, 2018).

Furthermore, the host sensor IFI16 (or AIM2) that encounters viral determinants could also interact with the inflammasome and hence induce the pro-caspase-1 activation by an apoptosis-associated speck-like protein containing a caspase recruitment domain (Johnson et al., 2013). HSV-1 immediate early protein infected-cell polypeptide 0 (ICP0), which is an E3 ubiquitin ligase, has been shown to successfully inhibit IFI16 activation by guiding it to the proteasome and accelerating its degradation (Johnson et al., 2013). HSV viral protein 22 (VP22), encoded by the UL49 gene, has also been reported to block this pro-caspase-1 activation and inhibit the secretion of pro-inflammatory cytokines such as IL-1β or IL-8 (Maruzuru et al., 2018). Moreover, apart from directly inhibiting the activation of IFI16, a recent study revealed that an HSV-1 UL41 protein contributed to the decrease of IFI16 expression by degrading its mRNA (Orzalli et al., 2016).

In addition, within the nucleus, viral DNA is not only sensed but also loaded with heterochromatin to silence its expression and to restrict viral replication (Orzalli and Knipe, 2014). Several restriction factors, including IFI16 and promyelocytic leukemia (PML) protein, limit viral gene expression and replication (Orzalli et al., 2013; Merkl et al., 2018). Merkal et al. have defined a novel mechanism of epigenetic silencing of HSV-1 DNA, which revealed that an IFI16 filamentous structure could recruit other restriction factors, including PML protein, speckled protein 100 (Sp100), and alpha-thalassemia mental retardation syndrome x-linked (ATRX), to aid in the restriction (Merkl and Knipe, 2019). Notably, an increasing body of evidence suggests that HSV-1 ICP0 protein can promote the degradation of the IFI16, ATRX, Sp100, as well as PML proteins and prevent their restriction activities (Lukashchuk and Everett, 2010; Jurak et al., 2012; Orzalli et al., 2012). However, further studies are still needed to identify the full protein composition of this new infected cell nuclear structure and investigate the underlying mechanisms by which HSV-1 regulates the downstream pathways related to IFI16.

### Cyclic Guanosine Monophosphate-Adenosine Monophosphate Synthase-Stimulator of Interferon Genes

Several DNA viruses, including adenovirus, cytomegalovirus, hepatitis B virus, and HSV-1, can induce IFN-I in a cGAS/stimulator of interferon genes (STING)-dependent pathway (Gao et al., 2013; Ma et al., 2015; Wu et al., 2015; Dansako et al., 2016; Paijo et al., 2016). As a result, cGAS plays a crucial role in antiviral innate immunity, which triggers cyclic GMP-AMP (cGAMP) production through its enzymatic activity upon binding to cytosolic dsDNA (Cai et al., 2014). Then, cGAMP activates the endoplasmic reticulum (ER)-anchored STING, which then translocates from the ER to the Golgi apparatus and leads to the recruitment and phosphorylation of TANK-binding kinase 1 (TBK1) and IκB kinase (IKK). Notably, the trafficking step from the ER to the Golgi apparatus is crucial for the induction of IFN-I transcription by STING (Saitoh et al., 2009). Finally, the IRF3 and NF-κB are activated to induce the production of IFN-I and inflammatory cytokines (Sun et al., 2013; Wu et al., 2013).

At the upstream of the cGAS/STING signaling pathway, our previous study showed that an HSV-1 UL41 protein could degrade cGAS mRNA during viral infection (Su and Zheng, 2017). This finding showed that the ectopic expression of UL41 remarkably reduced the accumulation of cGAS *via* its RNase activity and downregulated the cGAS/STING-mediated activation of the IFN pathway to escape antiviral innate immune responses. Besides, HSV-1VP22, a highly abundant tegument protein, also has an effect on this DNA-sensing pathway. VP22 has been shown to interact directly with cGAS and thus suppress the enzymatic activity of cGAS, and it acts as an important inhibitor of IFN-β production and downstream antiviral genes (Huang et al., 2018).

Interestingly, Deschamps et al. have showed that the stable overexpression of HSV-1 tegument protein UL46 in cells can reduce the expression of STING and inhibit its downstream IFN-I signaling pathway (Deschamps and Kalamvoki, 2017). However, during viral infection, UL46 does not affect the expression and function of STING, which is obviously illogical. Our recent study revealed a novel underlying mechanism – that HSV-1 UL46 downregulated antiviral immune responses by interacting directly with TBK1. UL46 was shown to significantly reduce the dimerization of TBK1 and affect the interaction between TBK1 and IRF3, which resulted in inhibiting the activation of TBK1 and the production of IFN-β (You et al., 2019). Based on this evidence, HSV-1 UL46 disrupts the cGAS-STING signaling pathway and possibly interacts with both STING and TBK1 *via* separate domains.

An increasing amount of evidence has shown that post-translational modifications, such as phosphorylation and ubiquitination, directly or indirectly modulate the cGAS/STING pathway and significantly affect viral infections (Liu et al., 2013, 2016). cGAS can be targeted for deamidation by the HSV-1 tegument protein UL37, which causes cGAS inactivation and facilitates HSV-1 lytic replication (Zhang et al., 2018). Moreover, Sun et al. demonstrated that HSV-1 UL36 ubiquitin-specific protease (UL36USP) could inhibit viral capsid ubiquitination and subsequent degradation through its deubiquitylase activity, thus avoiding the recognition of cGAS instead of affecting the stability of cGAS or STING (Sun et al., 2015).

These findings reveal some novel mechanisms of viral evasion. More importantly, the multifaceted strategy of HSV-1 to compromise the DNA-sensing pathway highlights how STING is a key restriction factor for HSV-1.

#### TANK-Binding Kinase 1-Interferon Regulatory Factor 3

At the downstream of the cGAS/STING signaling pathway, studies from our lab demonstrated that VP24, a serine protease of HSV-1, could also block dsDNA-triggered IFN production by abrogating the interaction between TBK1 and IRF3 and inhibiting the activation of IRF3 (Zhang et al., 2016), while HSV-1 VP16 could prevent IRF3 from recruiting the CREB-binding protein (CBP) coactivator, thus blocking IRF3-mediated transcription (Xing et al., 2013). What is more, protein kinase US3 of HSV-1 has been shown to interact with and hyperphosphorylate IRF3 at Ser175 to prevent IRF3 activation and dampen IFN-I production (Wang et al., 2013, 2014). Christensen et al., found that HSV-1 ICP27 protein, a product of viral immediate early genes conserved among all human herpesviruses, could impair the upstream of IRF3 activation (but could not impair TBK1 phosphorylation) by interacting with TBK1 and STING in human macrophages (Christensen et al., 2016). Nevertheless, ICP27, as an immediate early gene, can regulate the production of many viral genes through stimulating transcription and translation of viral early and late genes, indicating that the results from the ICP27 deletion virus does not guarantee that the viral immune evasion is mediated by ICP27 (Sandri-Goldin, 2011). It is plausible that ICP27 might affect the IFN-I signaling pathway through the regulation of the expression of viral early and late genes. Altogether, these findings from our and other labs will be important for understanding the interaction between HSV-1 and the host DNA-sensing signal pathway.

#### TANK-Binding Kinase 1-Nuclear Factor κB

NF-κB is known for its critical role in innate immune responses and can be strongly induced at the downstream of most PRRs, resulting in the production of IFN-β as well as inflammatory interleukins (Woronicz et al., 1997). During the activation of NF-κB, IκBs are phosphorylated by activated IKK, and then the NF-κB p50/p65 heterodimer is released and transferred to the nucleus, which finally regulates the innate immune responses (Bonizzi and Karin, 2004; Hayden and Ghosh, 2008; Chiu et al., 2009b; Iwai, 2014). New evidence suggests that TBK1 is essential for the activation of the NF-κB signaling pathway mediated by dsDNA and utilizes the IKK activation loop to activate the subunit p65 (Abe and Barber, 2014).

Consequently, HSV-1 has also evolved various elaborate mechanisms to subvert this signaling pathway. For example, the HSV-1 immediate early protein ICP0 interacts with NF-κB subunits p50/p65 and degrades p50 through its E3-ubiquitin ligase activity (Zhang et al., 2013a). Kim et al. also reported that HSV-1 ICP27 could repress NF-κB activity through blocking the phosphorylation and ubiquitination of IκBα and stabilize IκBα to evade immune responses during the very early period of HSV-1 infection (Kim et al., 2008). Meanwhile, our study has showed that HSV-1 UL36USP deubiquitinates IκBα and prevents its degradation, which inhibits p50/p65 transportation and finally abrogates NF-κB activation (Ye et al., 2017).

The production of IFN-I depends on transcription factors of both IRF3 and NF-κB, which bind to distinct regulatory domains in the promoter. HSV-1 US3 has been shown to hyperphosphorylate p65/RelA at serine 75, which significantly inhibited NF-κB activation by blocking its nuclear translocation and decreased the expression of inflammatory chemokine interleukin-8 (Wang et al., 2013, 2014). Similarly, HSV-1 UL42, a DNA polymerase processivity factor, also significantly prevents NF-κB-dependent gene expression by retaining p50/p65 in the cytoplasm (Zhang et al., 2013b). Additionally, HSV-1 UL24, another conserved viral protein that is important for viral replication, selectively blocked activation of the NF-κB, but not IRF3, by binding to Rel homology domains of p50/p65 and abrogating their nuclear translocation (Xu et al., 2017). For the first time, UL42 and UL24 are demonstrated to effectively inhibit cGAS/STING-induced NF-κB activation and dsDNA-mediated IFN-β or IL-6 production during HSV-1 infection. It is worth noting that some HSV-1 proteins may target the cytosolic DNA-sensing pathway through similar mechanisms.

### Janus Kinase-Signal Transducer and Activator of Transcription

Although HSV-1 can antagonize the production of IFN-I *via* many mechanisms, a certain amount of IFN-I produced during early infection will induce ISGs through the Janus kinase-signal transducer and activator of transcription (JAK-STAT). HSV-1 also evolves mechanisms to disrupt the JAK-STAT pathway, which is the downstream of the IFN signaling pathway, and further evades the antiviral immunity.

It is known that IFN-I can be initially produced following the detection of viral RNA, DNA, or proteins by intracellular PRRs in host cells (Raftery and Stevenson, 2017). IFN-I include IFN-α as well as IFN-β, IFN-ε, IFN-κ, and IFN-ω (van Pesch et al., 2004; Ivashkiv and Donlin, 2014). After its secretion, IFN-I interacts with the cell-surface receptor known as the type I IFN receptor (IFNAR), which is a heteromeric receptor that contains subunit IFNAR1 and IFNAR2 (Decker et al., 2005). When the receptor is activated, it recruits and phosphorylates the tyrosine kinase 2 (Tyk2) and JAK1, which leads to the formation of a heterodimer of phosphorylated STAT1 and STAT2 (Brooks et al., 2014). Then, STAT1/STAT2 binds to the cytoplasmic IRF9, forming a complex known as IFN-stimulated gene factor 3 (ISGF3) that translocates into the nucleus and binds to a DNA sequence called the IFN-stimulated response element (ISRE), resulting in the transcription of many ISGs, including viperin, zinc-finger antiviral protein (ZAP), Cholesterol 25-hydroxylase (Ch25h), tetherin, ISG15, and some proteins of the *tripartite motif* (TRIM) family, which are responsible for the effector properties of directly antiviral responses (Schoggins and Rice, 2011; Schneider et al., 2014; Raftery and Stevenson, 2017). However, HSV-1 has evolved multiple strategies to evade this process.

Johnson et al. observed that HSV-1 protein ICP27 was sufficient to inhibit IFN-mediated STAT1 phosphorylation and nuclear accumulation at or before the phosphorylation of JAK (Johnson et al., 2008; Johnson and Knipe, 2010). HSV-1 can also downregulate the protein levels of JAK1 and STAT2 through the virion host shutoff protein at a relatively high multiplicity of infection (Chee and Roizman, 2004). Previous studies from our and other labs have shown that UL41 can degrade the mRNAs of some ISGs, such as IFIT3, ZAP, viperin, tetherin, as well as Ch25h, to attenuate the IFN-mediated antiviral immune responses *via* its RNase activity (Zenner et al., 2013; Shen et al., 2014; Su et al., 2015; Jiang et al., 2016; You et al., 2017; Yuan et al., 2018). Furthermore, we have recently demonstrated that HSV-1 UL36USP also antagonizes the activation of the IFN-JAK-STAT pathway through specifically binding to IFNAR2 and blocking the interaction between JAK1 and IFNAR2, which is independent of its DUB activity (Yuan et al., 2018).

The HSV-1 latency-associated transcript (LAT), which is not known to encode a functional protein but regulate the virus latency and reactivation, has been shown to inhibit apoptosis *via* inhibiting activation of pro-apoptotic caspases and promoting cell survival or immune evasion (Perng et al., 2000; Henderson et al., 2002; da Silva and Jones, 2013; Phelan et al., 2017). However, the mechanism of this process is unknown. Tormanen et al. have recently observed that LAT affected apoptosis by downregulating the expression of JAK1 and JAK2, as well as several downstream ISGs of the JAK-STAT pathway at the level of a transcriptional mechanism during HSV-1 latency (Tormanen et al., 2019).

Overall, a growing amount of evidence suggests that HSV-1 has evolved multiple mechanisms to inhibit IFN signaling not only in infected cells but also in neighboring cells, thereby allowing for increased viral replication and spread. Therefore, the increased understanding of the IFN-JAK-STAT signal pathway is essential for our ambition to develop novel, less toxic, and more effective anti-viral treatments.

## Viral Manipulation of Other Antiviral Processes *Via* Regulating Tripartite Motif Proteins

The function of autophagy is well known for its regular degradation and recycling of cellular components through isolating certain targeted cytoplasmic proteins within a double-membraned autophagosome (Dong and Levine, 2013). The pathway of autophagy is an essential component of host defense against viral infection and innate immune responses (Lussignol and Esclatine, 2017). Besides, recent studies have demonstrated that autophagy and innate immune signaling, in particular the IFN-I signaling pathway, are intricately interconnected (Tal and Iwasaki, 2009; Levine et al., 2011; Deretic et al., 2013). It is worth noting that several key molecules, such as TBK1, IRF3, and p62, involved in IFN-β induction are also important regulators of autophagy (Sharma et al., 2003; Pilli et al., 2012; Richter et al., 2016).

Interestingly, TRIM proteins, which belong to the larger family of RING E3 ligases and are well known to regulate antiviral cytokine production in DNA-sensing pathways, also play important roles in autophagy as well as autophagy-mediated antiviral defenses (Versteeg et al., 2013; Mandell et al., 2014). Previous study indicated that HSV-1 infection could lead to ER stress-relating signaling networks including many pathways of immune responses and other mechanisms that restrict viral pathogenesis (Li et al., 2015). However, to survive and propagate within the host, many viruses, including HSV-1, have evolved a variety of strategies to evade autophagy for their own benefit.

It has been proved that both TRIM56 and TRIM32 could catalyze K63-linked polyubiquitination on STING when STING had been activated by cGAMP and then translocated from the ER to the Golgi apparatus (Tsuchida et al., 2010; Ishikawa and Barber, 2011; Zhang et al., 2012). Meanwhile, after activation of TBK1 and IRF3, this excess of STING can be degraded by p62/Sequestosome1-dependent autophagy (Prabakaran et al., 2018). Konstantin et al. have revealed that unconventional K27-linked auto-ubiquitination is essential for the GTP hydrolysis activity of TRIM23, which is necessary for the recruitment of TRIM23 to autophagosomal membranes and the activation of TBK1- and p62-mediated selective autophagy (Sparrer et al., 2017). Interestingly, HSV-1 US11 could drastically suppress this autophagy loop by disrupting the TRIM23-TBK1-Hsp90 complex and inhibiting the restriction of HSV-1 infection (Liu et al., 2018). The underlying mechanism is that US11 can block recruitment of TBK1 by targeting the C-terminal ADP-ribosylation factor domain in TRIM23, which results in a negative impact on both pathways of autophagy and the type I IFN response.

Many functions of HSV-1 ICP0 have been directly linked to its E3 ubiquitin ligase activity that is required for efficient infection (Lilley et al., 2010; Orzalli et al., 2012, 2013). Conwell et al. have presented that ICP0 utilized its own RING E3 ligase activity to induce polyubiquitination and degradation of TRIM27, which might play a role in intrinsic resistance to HSV-1 infection (Conwell et al., 2015). Similarly, the Epstein-Barr virus induces the expression of TRIM29, which was reported to modify STING with K48-linked polyubiquitin and negatively regulate innate immune responses to DNA viruses (Xing et al., 2017).

The results revealed some previously undocumented mechanisms of DNA viruses in infected cells and their resistance to innate immunity, which has greatly improved our understanding of the interplay between HSV-1 and host antiviral responses through targeting TRIM family.

## Conclusion and Perspectives

In this review, a growing number of findings have explained the active interactions between HSV-1 and the host antiviral innate immunity, which have revealed some novel mechanisms of viral evasion. Upon infection, HSV-1 has developed sophisticated strategies with viral proteins to counteract IFN-I production in innate immune responses, mainly through interactions with the DNA-sensor-mediated antiviral signal pathways. Through these achievements, we stand to gain an enriched understanding of viral evasion mechanisms in host cells. Nevertheless, there remain several knowledge gaps to be further investigated.

Firstly, how cGAS senses and binds to the dsDNA of HSV-1 has remained elusive. Secondly, the mechanisms through which the dysregulation of innate immune responses by HSV-1 affect human viral diseases and pathogenesis, such as AD, remain largely elusive. In other words, the clinical models of many observations coming from the overexpression of viral proteins in human cell systems remain to be established in the future. Thirdly, it warrants further investigation whether HSV-1 evades the antiviral potential of the TRIM family, and this will open up a new potential area of viral immune escape mechanisms.

Strikingly, there is still a firestorm of controversy about what is the DNA sensor of a virus in the nucleus really is. Finally, perhaps most challenging and essential issue is what nuclear receptor initiates the innate immune response to DNA viruses, how does it achieve this, and does it include HSV-1? Surprisingly, during preparation of our manuscript, a recent discovery from Cao’s group has showed that the nuclear-localized heterogeneous nuclear ribonucleoprotein A2B1 (hnRNPA2B1) recognizes viral DNA and then translocates to the cytoplasm where it activates the TBK1-IRF3 pathway and amplifies IFN-α/β production (Wang et al., 2019). But, whether hnRNPA2B1 plays an important role in initiating the IFN production and enhancing the cytoplasmic antiviral signaling in HSV-1 infection still needs further investigation. Further understanding of these questions will help us to reveal the detailed and molecular mechanisms of HSV-1 infection or viral diseases, which may accelerate future development of novel antiviral therapeutics and vaccines.

## Author Contributions

YL wrote the manuscript and designed the figures. CZ reviewed and modified the manuscript.

### Conflict of Interest

The authors declare that the research was conducted in the absence of any commercial or financial relationships that could be construed as a potential conflict of interest.
