# Corrigendum: A tug of war: DNA-sensing antiviral innate immunity and herpes simplex virus type I infection

**DOI:** 10.3389/fmicb.2023.1253103

**Published:** 2023-07-26

**Authors:** Yingying Lin, Chunfu Zheng

**Affiliations:** Department of Microbiology, Immunology and Infectious Diseases, University of Calgary, Calgary, AB, Canada

**Keywords:** herpes simplex virus type I, DNA sensor, immune evasion, interferon, antiviral immunity

In the published article, there was an error in **affiliation 2**.

Instead of:

“Yingying Lin^1^, Chunfu Zheng^1,2^

^“1^Department of Immunology, School of Basic Medical Sciences, Fujian Medical University, Fuzhou, China.

^2^Department of Microbiology, Immunology and Infectious Diseases, University of Calgary, Calgary, AB, Canada.”

The affiliation should be corrected as follows:

“Yingying Lin^1^, Chunfu Zheng^1^

^“1^Department of Microbiology, Immunology and Infectious Diseases, University of Calgary, Calgary, AB, Canada.”

The authors apologize for this error and state that this does not change the scientific conclusions of the article in any way. The original article has been updated.

